# Pharmacokinetic Analysis of an Oral Multicomponent Joint Dietary Supplement (Phycox^®^) in Dogs

**DOI:** 10.3390/pharmaceutics9030030

**Published:** 2017-08-18

**Authors:** Stephanie E. Martinez, Ryan Lillico, Ted M. Lakowski, Steven A. Martinez, Neal M. Davies

**Affiliations:** 1 Department of Veterinary Clinical Sciences, College of Veterinary Medicine, Washington State University, Pullman, WA 99164, USA; smartinez@vetmed.wsu.edu; 2 College of Pharmacy, Rady Faculty of Health Sciences, University of Manitoba, Winnipeg, MB R3E 0T5, Canada; umlillic@myumanitoba.ca (R.L.); ted.lakowski@umanitoba.ca (T.M.L.); 3 Comparative Orthopedic Research Laboratory, Department of Veterinary Clinical Sciences, College of Veterinary Medicine, Washington State University, Pullman, WA 99164, USA; martinez@vetmed.wsu.edu; 4 Faculty of Pharmacy and Pharmaceutical Sciences, University of Alberta, Edmonton, AB T6G 2H7, Canada

**Keywords:** glucosamine, nutraceutical, osteoarthritis, pharmacokinetics, polyphenols, veterinary medicine

## Abstract

Despite the lack of safety, efficacy and pharmacokinetic (PK) studies, multicomponent dietary supplements (nutraceuticals) have become increasingly popular as primary or adjunct therapies for clinical osteoarthritis in veterinary medicine. Phycox^®^ is a line of multicomponent joint support supplements marketed for joint health in dogs and horses. Many of the active constituents are recognized anti-inflammatory and antioxidant agents. Due to a lack of PK studies in the literature for the product, a pilot PK study of select constituents in Phycox^®^ was performed in healthy dogs. Two novel methods of analysis were developed and validated for quantification of glucosamine and select polyphenols using liquid chromatography-tandem mass spectrometry. After a single oral (PO) administrated dose of Phycox^®^, a series of blood samples from dogs were collected for 24 h post-dose and analyzed for concentrations of glucosamine HCl, hesperetin, resveratrol and naringenin. Non-compartmental PK analyses were carried out. Glucosamine was detected up to 8 h post-dose with a *T*_max_ of 2 h and *C*_max_ of 9.69 μg/mL. The polyphenols were not found at detectable concentrations in serum samples. Co-administration of glucosamine in the Phycox^®^ formulation may enhance the absorption of glucosamine as determined by comparison of glucosamine PK data in the literature.

## 1. Introduction

Osteoarthritis (OA) continues to present significant therapeutic problems in humans, equines, canines and other companion animals despite its clinical prevalence. Non-steroidal anti-inflammatory agents (NSAIDs) remain the most common therapies across species for attenuation of clinical signs of OA [1,2,3]. NSAIDs act by inhibiting cyclooxygenases, which are involved in the production of prostaglandins resulting in analgesic, anti-inflammatory and antipyretic effects [4,5]. Chronic use of NSAIDs is associated with adverse effects including gastrointestinal ulcers, liver toxicity, hemorrhaging and negative effects on chondrocytes and cartilage-matrix formation [1,4,5,6,7]. Due to the possible adverse effects of NSAIDs, there has been an interest in human and veterinary medicine to identify dietary supplements (nutraceuticals) that may serve as safer, efficacious alternatives or adjuncts to NSAIDs for the management of OA [8].

Presently, multicomponent formulations are a trend in dietary supplements and nutraceuticals with the notion that several chemical constituents may interact with multiple targets to evoke interdependent pharmacological activities to achieve optimal and potentially synergistic effects [9]. In regards to pharmacological and pharmacokinetic (PK) evaluations, multicomponent dietary supplements pose increased difficulty in comparison to a supplement containing a single chemical constituent; not only due to the increase in the amount of chemical constituents to monitor but also the increased potential for xenobiotic–xenobiotic interaction. Clearly, PK analysis is required on the major constituents present in these formulations; however, the reports of polyPK analyses of dietary supplements are lacking in the literature [9]. A comprehensive understanding of the absorption, distribution, metabolism and excretion of dietary supplements and natural health products is important for sustaining effective concentrations of active compounds at the sites of action but, unfortunately, many dietary supplements for both human and veterinary health do not undergo PK evaluation and are sold with dosing instructions without any scientific rationale [10]. The lack of PK evaluation for commercial dietary supplements is particularly evident in veterinary medicine as no federal testing requirements are in place despite the increased clinical use of these dietary supplements [11,12,13].

Joint health products such as glucosamine hydrochloride and chondroitin sulfate represent the largest category of dietary supplements in veterinary medicine. A novel product that falls into this category is the multicomponent Phycox^®^ product line produced by Dechra Veterinary Products (Overland Park, KS, USA). The Phycox^®^ line produces products formulated for dogs and horses marketed as a joint health supplement that supports joint mobility and healthy bone structure [14]. Table 1 contains the extensive list of active ingredients of Phycox^®^ soft chews for dogs. Many of the ingredients are not single chemical constituents but complex matrices, such as the Phycox^®^ active ingredient consisting of a proprietary blue-green algae extract containing biliprotein, c-phycocyanin, citrus bioflavonoids, grape seed extract and turmeric; all of which have been reported to possess anti-inflammatory and/or antioxidant properties in vitro, and, in some cases, in vivo [15,16,17,18,19,20,21,22,23]. Furthermore, flaxseed is a source of fatty acid omega-3, as alpha-linoleic acid (ALA) is a precursor to eicosapentaenoic acid (EPA) and docosahexaenoic acid (DHA), each of which could also have potential bioactivity and therapeutic properties. Additionally, a recent study from our group demonstrated that many Phycox^®^ constituents as well as the whole preparation possess cyclooxygenase-2 inhibitory activities and, in an in vitro model of canine OA, no significant difference between the Phycox^®^ preparation and the NSAID, carprofen, were found in the reduction of most OA biomarkers warranting further clinical investigations of Phycox^®^ [11]. It is entirely possible that the major components of the formulation including glucosamine, MSM, creatine, ALA, etc. may have a peripheral role.

**Figure 1:**
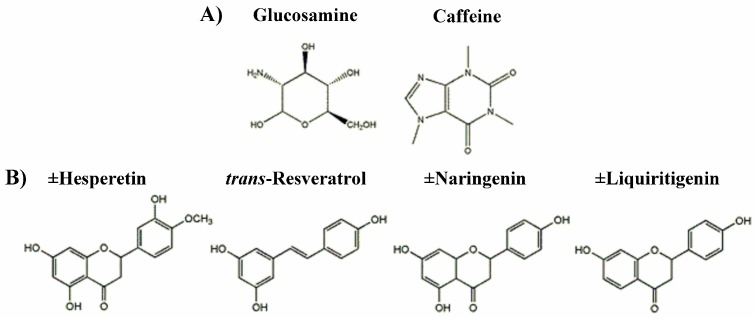
(**A**) Chemical structures of glucosamine and the internal standard caffeine; (**B**) chemical structures of ±hesperetin, ±naringenin, *trans*-resveratrol and the internal standard ±liquiritigenin.

To our knowledge, there are no studies published evaluating the PK disposition or the in vivo metabolism of the constituents of Phycox^®^ when administered via the Phycox^®^ soft chew in the dog. Individual PK studies in dogs exist for the following Phycox^®^ constituents: glucosamine [1,13], resveratrol (found in grape seed extract) [24] and naringenin (found in citrus bioflavonoids) [25,26]. The objectives of this study were to develop and validate the methods of analysis using liquid chromatography-tandem mass spectometry (LC-MS/MS) for glucosamine (Figure 1A), *trans*-resveratrol (Figure 1B), ±naringenin (Figure 1B) and ±hesperetin (component of citrus bioflavonoids) (Figure 1B), to quantify *trans*-resveratrol, ±naringenin and ±hesperetin in Phycox^®^ soft chews and to describe the pharmacokinetics of glucosamine, *trans*-resveratrol, ±naringenin, and ±hesperetin following administration of a single Phycox^®^ soft chew to female Beagle dogs in a pilot study.

## 2. Materials and Methods

### 2.1. Chemicals and Reagents

Phycox^®^ canine chewable whole tablets and the individual components, glucosamine hydrochloride, grape seed extract, citrus bioflavonoids, and Phycox^®^ premix were provided by Dechra Veterinary Products. ±Naringenin, ±hesperidin, ±liquiritigenin, caffeine, *trans*-resveratrol, β-glucuronidase type IX A (β-glucuronidase), were purchased from Sigma-Aldrich (St. Louis, MO, USA). β-Glucosidase from almonds was purchased from Tokyo Chemical Industry Co., Ltd. (Tokyo, Japan). Formic acid was purchased from Acros Organics (Geel, Belgium). HPLC-grade acetonitrile and methanol were purchased from EMD Millipore (Gibbstown, NJ, USA). Ultrapure water from a Milli-Q water system was used (Millipore, Billerica, MA, USA).

### 2.2. Analytical System and Conditions

The LC-MS/MS system used (Shimadzu, Kyoto, Japan) was connected to the liquid chromatography portion consisting of two Nexera™ LC-30AD pumps, a Nexera™ SIL-30AC auto injector, a CMB-2-A Prominence system controller, a DGU-20A5R degassing unit and a CT0-20A Prominence column oven. Data analysis was accomplished using Shimadzu LabSolutions (Version 5.3) software.

The LC-MS/MS was operated in DUIS mode (electrospray ionization and atmospheric pressure chemical ionization) using multiple reaction monitoring (MRM). The LC-MS/MS conditions consisted of a curved desolvation line temperature of 250 °C and heating block temperature of 400 °C. Nebulizing gas we delivered at 2 L/min and drying gas was delivered at 15 L/min.

### 2.3. Glucosamine

The analytical system described above was used for serum glucsamine concentration analysis. The analytical column used was a Primesep 200 (3.0 μm, 2.1 × 100 mm) (SIELC Technologies, Wheeling, IL, USA) mixed function cation exchange column. The mobile phase consisted of a pH gradient with mobile phases A (0.05% aqueous formic acid) and B (1% formic acid) in 50% aqueous acetonitrile. The gradient was as follows: 0% B for 3 min, linear gradient from 0% to 80% B in 2.5 min, linear gradient from 80% to 100% B in 0.1 min, and from 100% to 0% B in 1 min. The column equilibrated for 3 min at 0% B prior to the next injection. Separation was carried out at 35 ± 0.5 °C with a flow rate of 0.4 mL/min. Both glucosamine and the internal standard, caffeine, were monitored in positive mode. Table 2 describes the parent and daughter ion mass to charge ratios (*m*/*z*) used along with the collision energy of each compound.

### 2.4. Polyphenols

The analytical system described above was used for serum polyphenol analysis. The analytical column used was a Waters ACQUITY UPLC^®^ BEH C_18_ column (3.0 μm, 2.1 × 100 mm) (Waters Corporation, Milford, MA, USA). An isocratic mobile phase consisting of 50% aqueous methanol with 0.1% formic acid was employed. Separation was carried out at 40 ± 0.5 °C with a flow rate of 0.4 mL/min. ±Liquiritigenin was used as the internal standard. All compounds were monitored in positive mode. Table 2 describes the parent and daughter ion mass to charge ratios (*m*/*z*) used along with the collision energy of each compound.

### 2.5. Stock and Working Standard Solutions

Methanolic stock solutions of glucosamine, caffeine, ±hesperetin, ±naringenin, *trans*-resveratrol and ±liquiritigenin were prepared at 100 μg/mL concentrations. These solutions were protected from light and stored at −20 °C between uses for no longer than 3 months. Calibration standards in serum and methanol were prepared from stock solutions by sequential dilution, yielding a series of concentrations; 0.005, 0.01, 0.05, 0.1, 0.5, 1.0, 5.0, 10 and 20 μg/mL for glucosamine, ±hesperetin, ±naringenin and *trans*-resveratrol. Quality control (QC) samples were prepared from stock solutions by dilution to yield target concentration of 0.075, 1.5, and 15 μg/mL.

### 2.6. Sample Preparation for Standard Curves

#### 2.6.1. Glucosamine

The working standards of glucosamine were added to blank dog serum (100 μL) in microcentrifuge tubes to achieve the desired final concentration previously described along with 40 μL of internal standard, caffeine (diluted to a concentration of 0.1 μg/mL from stock solution). One milliliter of cold acetonitrile (−20 °C) was added to the samples to precipitate proteins. The samples were vortexed and centrifuged at 20,160× *g* for 5 min. The supernatant was transferred to new microcentrifuge tubes and evaporated to dryness using a Savant SPD1010 SpeedVac Concentrator (Thermo Fisher Scientific, Inc., Asheville, NC, USA). The residues were reconstituted with 50 μL of starting mobile phase (0.05% aqueous formic acid), vortexed for 30 seconds and centrifuged at 20,160× *g* for 5 min. The supernatants were transferred to 30 kDa centrifugal filter tubes and centrifuged for 30 min at 9000× *g*. The filtrate was transferred to HPLC vials and 5 μL were injected into the LC-MS/MS system.

#### 2.6.2. Polyphenols

The working standards of ±hesperetin, ±naringenin and *trans*-resveratrol were added to blank dog serum (100 μL) in microcentrifuge tubes to achieve the desired final concentration previously described along with 20 μL of internal standard, ±liquiritigenin (diluted to a concentration of 10 μg/mL from stock solution). One milliliter of cold acetonitrile (−20 °C) was added to the samples to precipitate proteins. The samples were vortexed and centrifuged at 20,160× *g* for 5 min. The supernatant was transferred to new microcentrifuge tubes and evaporated to dryness using a Savant SPD1010 SpeedVac Concentrator. The residues were reconstituted with 50 μL of mobile phase (50% aqueous methanol with 0.1% formic acid), vortexed for 30 seconds and centrifuged at 20,160× *g* for 5 min. The supernatants were transferred to 30 kDa centrifugal filter tubes and centrifuged for 30 min at 9000× *g*. The filtrate was transferred to HPLC vials and 5 μL were injected into the LC-MS/MS system.

### 2.7. Precision, Accuracy and Recovery

The inter-day precision and accuracy of the two assays were determined from the results of three replicate assays on three different days during a one-week period. Three standard curves (0.001–20 μg/mL) with the inclusion of the 3 QC sample concentrations (0.075, 1.5, and 15 μg/mL) for each assay were used. The precision of the assays was evaluated by the coefficient of variations (CV) of the QC samples. The accuracies were determined by the mean percentage error of measured concentrations to the expected concentrations (percent bias) of the QC samples using the constructed standard curves.

Recoveries of glucosamine and the polyphenols at the QC sample concentrations were determined. Recovery was assessed by the comparing the measured values of QC samples in methanol to theoretical values of QC samples.

### 2.8. Content Analysis of Polyphenols in Phycox^®^

The amount of glucosamine in each Phycox^®^ soft chew is known and listed under the active ingredients on the packaging. However, specific amounts of ±hesperetin, ±naringenin and *trans*-resveratrol contained in the citrus bioflavonoids and grape seed extract ingredients, respectively, are unknown. To determine the amount of ±hesperetin, ±naringenin and *trans*-resveratrol in one Phycox^®^ soft chew, content analyses of the whole soft chew, the premix and individual ingredient constituents of grape seed extract and citrus bioflavonoids were undertaken. The polyphenol extraction procedure from dietary supplements has previously been described [27]. Briefly, three samples of soft chews from different lots were weighed and then pulverized. The premix, grape seed extract and citrus bioflavonoids were already in powdered form. One gram of each product was weighted and placed in 15 mL tubes. Methanolic extractions were performed by adding 4 mL of methanol to each tube and then placing them on a rocking platform shaker at room temperature (23 ± 1 °C) for 3 h. Tubes were centrifuged for 10 min at 5000× *g*. The supernatants were collected in duplicate 100 μL aliquots (aglycone and total). The total samples were dried to completion using a Savant SPD1010 SpeedVac Concentrator and then reconstituted with phosphate-buffered saline (200 μL at pH 7.4). Twenty microliters of β-glucosidase (750 U/mL in phosphate buffered saline at pH 7.4) was added and samples were incubated for 48 h at 37 °C in a shaking incubator. β-Glucosidase acts by cleaving the glycosidic sugar moieties frequently present in plant extracts as previously described [28]. Cold acetonitrile (1 mL, 23 ± 1 °C) was added to stop the enzymatic reaction. To both sets of samples, the internal standard, 20 μL of 10 μg/mL ±liquiritigenin in methanol, was added. Samples were centrifuged at 20,160× *g* for 5 min. The supernatants were collected and dried to completion using a Savant SPD1010 SpeedVac Concentrator. Samples were reconstituted in mobile phase (50 μL of 50% aqueous methanol with 0.1% formic acid), vortexed and centrifuged at 20,160× *g* for 5 min. The supernatants were transferred to 30 kDa centrifugal filter tubes and centrifuged for 30 min at 9000× *g*. The filtrate was transferred to HPLC vials and 5 μL were injected into the LC-MS/MS system.

### 2.9. Animals and Compliance with Ethical Standards

The animal research protocol used in this study was reviewed and approved by the Institutional Animal Care and Use Committee at Washington State University (protocol #04224-011, approved 21 December 2014). Four commercially available purpose-bred intact female research Beagles from an Association for Assessment and Accreditation of Laboratory Animal Care (AALAC) certified source were used for the study. All dogs were 2 years of age at the onset of the study with a weight of 9.25 ± 0.81 kg (mean ± SD; range 8.4–10.2 kg). Animals were housed in AALAC approved facilities for animal research for 80 days acclimating to their environment prior to the initiation of the study. All dogs were fed a standard canine formulation (Proactive Health™ Adult Minichunks, IAMS, Mason, OH, USA) ration twice daily and housed in a temperature and humidity controlled room; 21 °C and 30%, respectively, while under 12 h light cycle conditions. The dogs underwent daily examinations and weekly body weight assessments. All animals were also given daily enrichment activities before, during, and after the study. At study termination all animals were transferred to an unrelated study.

### 2.10. Pharmacokinetic Studies

Dogs were fasted 12 h prior to the study but provided free access to water. Each subject was administered a single Phycox^®^ soft chew and a series of blood samples (3 mL) were collected at 0, 1, 2, 4, 6, 8, 12 and 24 h post-dose via saphenous or cephalic vein depending on the temperament of the animals. Animals were fed after 2 h post-dose. Following centrifugation of the blood samples, serum was removed and stored at −80 °C until analyzed.

### 2.11. Treatment of Pharmacokinetic Samples

#### 2.11.1. Glucosamine

One hundred microliters of serum from each PK sample was placed into a microcentrifuge tube. To all samples, except 0 h, 40 μL of 0.1 μg/mL internal standard stock solution (caffeine) was added along with 1.0 mL of cold acetonitrile (−20 °C) to precipitate proteins. The samples were vortexed and centrifuged at 20,160× *g* for 5 min. The supernatant was transferred to new microcentrifuge tubes and evaporated to dryness using a Savant SPD1010 SpeedVac Concentrator. The residues were reconstituted with 50 μL of starting mobile phase (0.05% aqueous formic acid), vortexed and centrifuged at 20,160× *g* for 5 min. The supernatants were transferred to 30 kDa centrifugal filter tubes and centrifuged for 30 min at 9000× *g*. The filtrate was transferred to HPLC vials and 5 μL were injected into the LC-MS/MS system.

#### 2.11.2. Polyphenols

The PK serum samples were separated into two sets of microcentrifuge tubes (total and aglycone) with 100 μL of serum in each. To the total samples, 20 μL β-glucuronidase (500 U/mL in 6.8 pH phosphate buffer) was added and incubated for 2 h at 37 °C [28]. To all samples except 0 h, 20 μL of 10 μg/mL internal standard stock solution (±liquiritigenin) was added along with 1.0 mL of cold acetonitrile (−20 °C) to precipitate proteins. The supernatant was transferred to new microcentrifuge tubes and evaporated to dryness using a Savant SPD1010 SpeedVac Concentrator. The residues were reconstituted with 50 μL of mobile phase (50% aqueous methanol with 0.1% formic acid), vortexed and centrifuged at 20,160× *g* for 5 min. The supernatants were transferred to 30 kDa centrifugal filter tubes and centrifuged for 30 min at 9000× *g*. The filtrate was transferred to HPLC vials and 5 μL were injected into the LC-MS/MS system.

### 2.12. Pharmacokinetic Analysis

PK samples were analyzed using Phoenix WinNonlin software (ver. 6.3; Certara, St. Louis, MO, USA) to derive the pharmacokinetic parameters for each individual dog and expressed as mean ± SEM. A non-compartmental analysis was used to calculate the PK parameters including area under the curve (AUC_0–∞_), the peak serum concentration (*C*_max_), time to reach peak serum concentration (*T*_max_), the apparent volume of distribution (Vd/F), the elimination half-life (*t*_1/2_), and the apparent clearance (CL/F). The elimination rate constant (KE), was estimated by log-linear regression of the serum concentrations. The AUC_0–∞_ was calculated using the long-linear trapezoidal rule for data from time of dosing to the last measured concentration plus the quotient of the last measured concentration divided by KE/2.303. The concentration time points of the animals were subjected to non-compartmental modeling. The aglycone concentrations of ±hesperetin, ±naringenin and *trans*-resveratrol were used for modeling.

### 2.13. Data Analysis

Compiled data were presented as mean and standard error of the mean (mean ± SEM). Where possible, the data were analyzed for statistical significance using SigmaPlot software (v. 13.0, SystatSoftware, Inc., San Jose, CA, USA). Student’s t-test was employed for unpaired samples to compare means between two groups, with a value of *p* < 0.05 considered statistically significant. The quantifications of concentrations were based on calibration curves constructed using the peak area ratio (PAR) of glucosamine and the polyphenols to internal standards, against the concentrations of glucosamine and the polyphenols using unweighted least squares linear regression.

## 3. Results

### 3.1. LC-MS/MS Analyses

#### 3.1.1. Glucosamine

**Figure 2:**
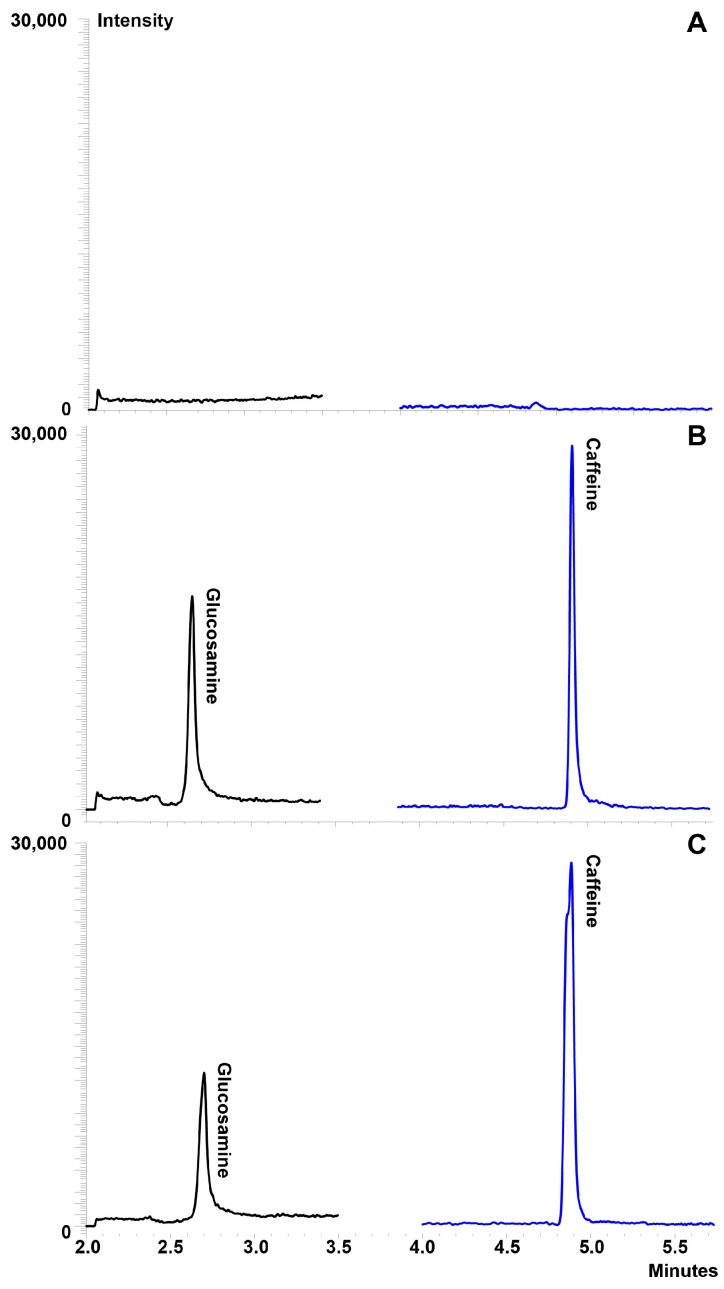
Representative chromatograms of glucosamine in serum: (**A**) blank serum demonstrating no interfering peaks co-eluted with the compounds of interest; (**B**) serum containing glucosamine and the internal standard at glucosamine at 10 μg/mL; and (**C**) pharmacokinetic sample at 2 h post-dose (450 mg glucosamine HCl via administration of one Phycox^®^ soft chew).

Various compositions of mobile phase and columns were tested to achieve the best resolution of glucosamine. Optimal separation was achieved with the combination of a Primesep 200 mixed function cation exchange column and pH gradient mobile phase consisting of: (A) 0.05% aqueous formic acid; and (B) 1% formic acid, in 50% aqueous acetonitrile with the following gradient: 0% B for 3 min, linear gradient from 0% to 80% B in 2.5 min, linear gradient from 80% to 100% B in 0.1 min, and from 100% to 0% B in 1 min. Following the gradient, the column was equilibrated for 3 min at 0% B prior to the next injection. Separation was best achieved using a flow rate of 0.4 mL/min and maintaining the column 35 ± 0.5 °C. Caffeine was selected as the internal standard owing to its similar chromatographic behavior and ionization efficiency to glucosamine. No interfering peaks co-eluted with the peaks of interest (Figure 2A). The retention times for glucosamine and caffeine were 2.7 min and 4.9 min, respectively, in serum (Figure 2B,C).

#### 3.1.2. Polyphenols

**Figure 3:**
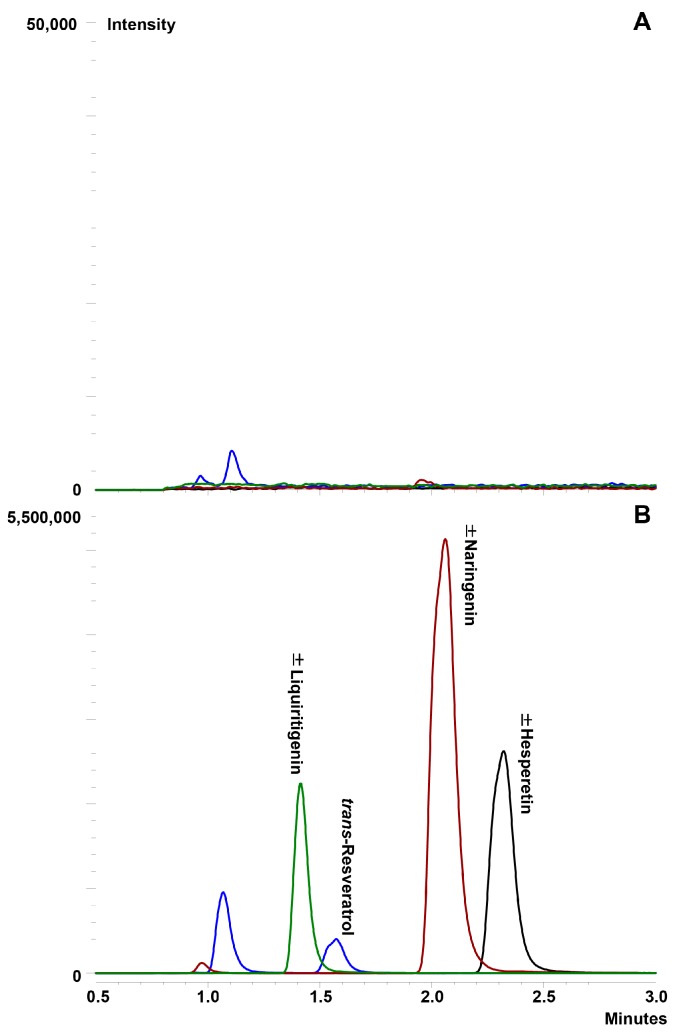
Representative chromatograms of ±hesperetin, trans-resveratrol and ±naringenin in serum: (**A**) blank serum demonstrating no interfering peaks co-eluted with the compounds of interest; and (**B**) serum containing polyphenols (20 μg/mL) and the internal standard.

As with glucosamine, various compositions of mobile phases and columns were investigated to achieve the best resolution of the compounds. Optimal separation was achieved using a simple, reverse-phase isocratic mobile phase consisting of 50% aqueous methanol with 0.1% formic acid with a flow rate of 0.4 mL/min. Separation of the compounds was achieved using a Waters ACQUITY UPLC^®^ BEH C_18_ column at 40 ± 0.5 °C. ±Liquiritigenin was used as the internal standard due to its structural similarity to the compounds of interest. No interfering peaks co-eluted with the peaks of interest (Figure 3A). The retention times for ±hesperetin, *trans*-resveratrol, ±naringenin and ±liquiritigenin were 2.3 min, 1.6 min, 2.1 min and 1.4 min, respectively (Figure 3B).

### 3.2. Linearity and Limit of Quantification

#### 3.2.1. Glucosamine

Calibration curves for glucosamine were linear from 5 ng/mL to 20 μg/mL in serum. The calibration curves showed good coefficient of determination (*R*^2^) > 0.95. The limit of quantitation for the method was 5 ng/mL. The limit of detection for the method was calculated by measuring the average background noise in blank samples at the retention time of the peaks of interest. Limit of detection was taken to be five times the background response and determined to be 1 ng/mL.

#### 3.2.2. Polyphenols

Calibration curves for ±hesperetin, *trans*-resveratrol and ±naringenin were linear from 5 ng/mL to 20 μg/mL in serum. The calibration curves for each compound showed good coefficient of determination (*R*^2^) > 0.99, (*R*^2^) > 0.97 and (*R*^2^) > 0.99 for ±hesperetin, *trans*-resveratrol and ±naringenin, respectively. The limits of detection for all three compounds were 5 ng/mL. The limits of detection for the method were calculated by measuring the average background noise in blank samples at the retention times of the peaks of interest and then limit of detection were taken to be five times the background response. Limit of detection for all compounds was found to be 1 ng/mL.

### 3.3. Precision, Accuracy and Recovery

#### 3.3.1. Glucosamine

The LC-MS/MS assay showed excellent accuracy and interday precision with a bias < 20% for the low QC sample (0.075 μg/mL) and < 3% for the medium (1.5 μg/mL) and high (15 μg/mL) QC samples along with CVs between 2.93 and 12.2% for the QC sample evaluated (Table 3). The recovery (extraction efficiency) of the assay was determined to be 34% in serum. These data indicate that the developed LC-MS/MS method for glucosamine quantitation is accurate, precise and reproducible.

#### 3.3.2. Polyphenols

The LC-MS/MS assay showed excellent accuracy and inter-day precision for all three compounds. The bias for ±hesperetin was < 15% for all QC concentrations and the CV was between 0.662 and 7.40% (Table 3). The bias for *trans*-resveratrol was < 18% for the low QC sample (0.075 μg/mL) and < 3% for the medium (1.5 μg/mL) and high (15 μg/mL) QC samples. The CVs for resveratrol were between 0.267% and 7.49%. The recovery (extraction efficiency) of the assay was determined to be 54.8%, 62.1% and 54.3% in serum for ±hesperetin, *trans*-resveratrol and ±naringenin, respectively. Bias for ±naringenin was < 12% for all QC samples and the CVs were between 2.78% and 11.4%. These data (Table 3) indicate that the developed LC-MS/MS method is accurate, precise and reproducible for all three compounds.

#### 3.3.3. Content Analysis of Polyphenols in Phycox^®^

The LC-MS/MS method of analysis for ±hesperitin, *trans*-resveratrol and ±naringenin was successfully applied to the determination and quantification of the compounds in Phycox^®^ soft chews, premix as well as the constituents; citrus bioflavonoids and grape seed extract. It was determined that ±hesperitin, *trans*-resveratrol and ±naringenin are present as the aglycones and not a mixture of aglycones and glycosides as determined by the lack of differences in concentrations between aglycone and total (incubated with β-glucosidase to cleave sugar moieties) samples. ±Hesperitin was determined to be the most prevalent of the three polyphenols in the premix, citrus bioflavonoids and Phycox^®^ soft chews. The concentration of ±hesperidin in the citrus bioflavonoids constituent was determined to be 362 μg/g and 80.8 μg/g in the Phycox^®^ premix. It was determined that a single Phycox^®^ soft chew contains 36.1 ± 5.39 μg of ±hesperetin. ±Naringenin was found at much lower concentrations than ±hesperetin in all of the samples. ±Naringenin was found to contain 68.6 μg/g and the Phycox^®^ premix was found to contain 6.77 μg/g ±naringenin. A single Phycox^®^ soft chew was determined to contain 2.88 ± 0.280 μg of naringenin. *Trans*-resveratrol was found at the lowest concentration in all samples of the three polyphenols measured. It was determined that the grape seed extract ingredient contains 0.681 μg/g of *trans*-resveratrol and the Phycox^®^ premix contains 0.482 μg/g. A single Phycox^®^ soft chew was found to contain 0.554 ± 0.177 μg of *trans*-resveratrol. The amount of grape seed extract and citrus bioflavonoids in each Phycox^®^ soft chew is proprietary information and not disclosed on the label claims.

### 3.4. Glucosamine

**Figure 4:**
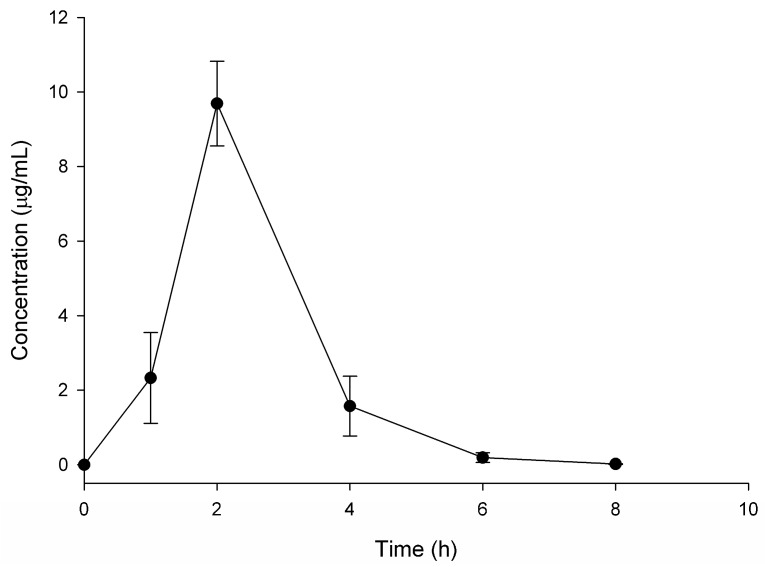
Glucosamine disposition in serum following oral administration of one Phycox^®^ soft chew containing 450 mg of glucosamine hydrochloride (*n* = 4, mean ± SEM).

The LC-MS/MS method of analysis for glucosamine was successfully applied to the determination and quantification of glucosamine in canines following administration of a single Phycox^®^ soft chew containing 450 mg of glucosamine hydrochloride. At time zero, no detectable concentrations of glucosamine were found. The mean serum concentration over time profile for glucosamine is shown in Figure 4. The pharmacokinetic parameters for glucosamine were calculated using non-compartmental analysis and are summarized in Table 4. These data indicate following PO administration of the Phycox^®^ soft chew, glucosamine was absorbed slowly into the systemic circulation with an average *T*_max_ of 2 h and an average *C*_max_ of 9.69 μg/mL, followed by elimination during the next 8 h, after which serum concentrations were below quantifiable concentrations (5 ng/mL). The apparent *t*_1/2_ of glucosamine after PO administration of the Phycox^®^ soft chew was approximately 35 min.

### 3.5. Polyphenols

±Hesperetin, *trans*-resveratrol and ±naringenin were not detected at quantifiable concentrations as either the aglycone or glucuronidated metabolite. Therefore, no pharmacokinetic parameters could be derived.

## 4. Discussion

Although the developed LC-MS/MS method for the quantification of ±hesperetin, *trans*-resveratrol and ±naringenin with a limit of quantification of 5 ng /mL proved to be sensitive, accurate and reproducible, when applied to the quantification of the compounds in canines following PO administration of a single Phycox^®^ soft chew containing 36.1 ± 5.39 μg of ±hesperetin, 0.554 ± 0.177 μg of *trans*-resveratrol and 2.88 ± 0.280 μg of ±naringenin, no compounds were detected at quantifiable concentrations as either the aglycone or glucuronidated metabolite. This is not unexpected given the extremely small quantities of these compounds administered via one Phycox^®^ soft chew and the generally low bioavailability of polyphenols in mammals [29,30]. Presently, there is no information on the bioavailability ±naringenin, ±hesperetin or *trans*-resveratrol in the literature in dogs. As the amounts of racemic flavonoids were so small no attempts were made to examine stereochemistry although we possess validated methods to do so in follow up studies if quantifiable in serum or urine [31,32,33].

Two previous studies have evaluated the oral pharmacokinetics of glucosamine HCl in Beagle dogs. Adebowale et al. [1] administered 1500–2000 mg of glucosamine HCl to eight dogs and used non-compartmental modeling to derive PK parameters. They report *T*_max_ values of glucosamine between 1.1 and 1.6 h and *C*_max_ values between 7.1 and 12.1 μg/mL [1]. The serum-concentration time curve reported in Adebowale et al. [1] was similar in shape to the curve reported in this study but the half-lives were reported to be between 1.52–2.4 h, which is 3–5 times longer than the half-live reported in this study [1]. After 4 h post-dose, Adebowale et al. [1] was not able to detect glucosamine [1]. The method employed by the study utilized a validated HPLC method with a limit of quantification of 1.25 μg/mL and involved pre-column derivatization [1]. The amount of glucosamine administered in the present study was 3.3 to 4.4 less than those administered in the study by Adebowale et al. [1] but the method employed in this study was 250 times more sensitive allowing for detection of much smaller concentrations of glucosamine. Various LC-MS-MS assays are validated in the literature across various fluids and species [33,34,35]. The current assay performance using 100 µL of serum is analytically comparable and robust. The second report of glucosamine HCl PK in dogs administered 1000 mg of glucosamine HCl to four male dogs using two commercially available chewable tablet formulations [12]. *T*_max_ and *C*_max_ values were reported to range 4.2–5.0 h and 1.8–2.6 μg/mL, respectively. The serum-concentration time curves reported by Maxwell et al. [12] were formulation dependent. The time curves generated by Maxwell et al. featured clear secondary peaks for two tablet products but not for the solution administered. A secondary peak has previously been reported in PO glucosamine HCl disposition in rats [32]. *t*_1/2_ was not reported by Maxwell et al. [12] At 12 h post-dose, Maxwell et al. was not able to detect glucosamine [12]. The method employed by the study used a modified HPLC method with a limit of quantification of 0.05 μg/mL (10 fold higher limit of quantitation than the method presented in this study) and involved pre-column derivatization [12]. To our knowledge, this is the first report of glucosamine PK in fasted dogs following PO administration of a multicomponent dietary supplement.

Administering glucosamine via a Phycox^®^ soft chew may enhance the absorption of glucosamine compared to previous studies where glucosamine HCl was administered alone or in conjunction with chondroitin sulfate [1,12]; however, this is not the first observation in the literature of a change in glucosamine disposition when co-administered with another compound besides chondroitin sulfate to dogs. Quian et al. [34] found that when glucosamine was co-administered with chitosan to Beagle dogs, absorption of glucosamine was increased compared to absorption of glucosamine administered alone likely due to chitosan’s ability to reversibly act on the epithelial tight junction to increase the paracellular permeability of glucosamine across the mucosal epithelial [34]. While chitosan is not present in Phycox^®^, it may be possible that other components of Phycox^®^ are able to increase glucosamine transcellular or paracellular permeability across the mucosal epithelial thereby enhancing absorption; however, it is also common that the pharmacokinetics of a given compound in a multi-component mixture may be significantly different from that of the single compound due to drug-drug interactions [9,36,37] With the plethora of chemical constituents in Phycox^®^ it is feasible that several constituents are interacting with multiple targets to increase absorption of glucosamine. The importance of fasting and gender on the pharmacokinetics of glucosamine remains to be further delineated.

## 5. Conclusions

Two method of analysis to detect glucosamine, ±hesperetin, *trans*-resveratrol and ±naringenin in dog serum were developed using a LC-MS/MS. These methods were found to be sensitive, reproducible and accurate. The methods were applied to a single-dose PK study of the multicomponent dietary supplement, Phycox^®^. Additionally, the method for quantification of ±hesperetin, *trans*-resveratrol and ±naringenin was employed to determine the amount of each compound in a single dose of Phycox^®^ as it is not disclosed on the label claim. No polyphenols at quantifiable concentrations were found as aglycones or glucuronide metabolites in the PK samples; however, glucosamine was successfully quantified using a novel method which requires no pre-column derivatization allowing PK parameters to be derived. The PK disposition of glucosamine was characterized by a slow absorption and a rapid terminal *t*_1/2_ of almost 35 min. While our PK parameters were at variance from previously reported PK parameters of glucosamine in fed Beagle dogs, the analytical method employed in this current study possessed greater sensitivity than previous studies. Future studies to determine the influence of gender and fasting and the mechanism whereby glucosamine absorption is increased by Phycox^®^ constituents as low glucosamine bioavailability may be considered a limiting factor for its therapeutic utility and improved absorption may result in increased efficacy for the clinical management of OA. Additionally, future studies should investigate the multiple-dosing PK of the constituents of Phycox^®^ as well as the PK of other bioactive constituents of the dietary supplement in both canines and equines since oral bioavailability and PK play an important role in the optimization of OA therapeutics for the management of clinical OA in people and other animals.

## Figures and Tables

**Table 1:** Phycox^®^ soft chew formula for canines (Pieloch 2006) (Phycox^®^ active ingredient is a proprietary blue-green algae extract).

Active Ingredients Per Soft Chew	
Glucosamine hydrochloride	450 mg
Methylsulfonylmethane	400 mg
Creatine monohydrate	250 mg
Alpha-linolenic acid	200 mg
Proprietary blend of citrus bioflavonoids, calcium phosphate, manganese sulfate, ascorbic acid (vitamin C), zinc sulfate, alpha lipoic acid, and grape seed extract	132 mg
Turmeric	50 mg
Phycox active	30 mg
Eicosapentaenoic acid (EPA)	9 mg
Docosahexaenoic acid (DHA)	6 mg
Boron	100 μg
Selenium	10 μg
Alpha tocopheryl acetate (vitamin E)	25 IU
Inactive ingredients	
Flaxseed oil, hydrolyzed vegetable protein, magnesium stearate, marine lipid concentrates, natural liver flavor, and sucrose	

**Table 2:** Mass spectral multiple reaction monitoring data for glucosamine, ±hesperetin, ±naringenin, *trans*-resveratrol and the internal standards caffeine and ±liquiritigenin.

Compound	Parent Ion *m/z*	Daughter Ion *m/z*	Collision Energy (eV)
Glucosamine	180.20	162.10	10
Caffeine (internal standard)	194.90	138.00 42.00 110.15	19 36 24
±Hesperetin	302.80	153.10	24.0
±Naringenin	273.90	153.10	24.0
*trans*-Resveratrol	228.80	135.10 107.20	13.0 23.0
±Liquiritigenin (internal standard)	257.90	137.0	−22.0

**Table 3:** Accuracy and precision of the LCMS quantitative analysis of glucosamine, ±hesperetin, *trans*-resveratrol and ±naringenin (*n* = 3).

Analyte	Nominal Value (μg/mL)	Measured Value (μg/mL) (Mean ± SEM)	CV (%)	Bias (%)
Glucosamine				
Low	0.075	0.063 ± 0.000	2.93	−16.4
Medium	1.5	1.46 ± 0.102	12.2	−2.91
High	15	14.6 ± 0.989	11.7	−2.45
±Hesperetin				
Low	0.075	0.087 ± 0.000	0.662	14.2
Medium	1.5	1.44 ± 0.062	7.40	−3.64
High	15	13.7 ± 0.155	1.97	−9.87
*trans*-Resveratrol				
Low Medium High ±Naringenin Low Medium High	0.075 1.5 15 0.075 1.5 15	0.088 ± 0.000 1.47 ± 0.063 14.6 ± 0.219 0.083 ± 0.004 1.55 ± 0.102 14.3 ± 0.219	0.267 7.49 2.57 7.98 11.4 2.78	17.7 −2.87 −1.50 10.1 3.30 −2.28

**Table 4:** Pharmacokinetic parameters of glucosamine (450 mg) administered in one Phycox^®^ soft chew in the dog (*n* = 4).

Pharmacokinetic Parameter	Mean ± SEM
AUC_0–∞_ (µg·h/mL)	20.4 ± 2.34
Vd/F (L/kg)	2.10 ± 0.254
CL/F (L/h/kg)	2.56 ± 0.446
*t*_1/2_ (h)	0.584 ± 0.034
*T*_max_ (h)	2.00 ± 0.000
*C*_max_ (μg/mL)	9.69 ± 1.14
